# Supplementary material for: Reconstruction of the transcriptional regulatory networks in the kidney of desert-adapted species
Source: Commun Biol. 2025 Nov 28;8:1719. doi: 10.1038/s42003-025-09124-2 (PMC12663168; doi:10.1038/s42003-025-09124-2)
Supplement: Supplementary file 1 — Supplementary Material [file 42003_2025_9124_MOESM1_ESM.pdf]

Reconstruction of the transcriptional regulatory networks in the kidney of desert-adapted species

*Fernando Alvira-Iraizoz [1, 2 *], Benjamin T. Gillard [1 *], Audrys G. Pauža [1], Panjiao Lin [1, 3], Alex Paterson [1], Pamela A. Burger [4], Mahmoud Hag Ali [5], Nabil Amor [6], Abdulaziz Alagaili [6], Abdu Adem [5 †], David Murphy [1 †], Michael P. Greenwood [1 †]*

[1] Molecular Neuroendocrinology Research Group, Bristol Medical School: Translational Health Sciences, University of Bristol, Dorothy Hodgkin Building, Bristol, United Kingdom.

[2] Genomics Medicine Unit, Navarrabiomed – Biomedical Research Centre, Pamplona, Spain.

[3] Double-crane Biotechnology Co., Ltd. Medical Science and Technology Centre, Zhongguancun Life and Science Park, Changping District, Beijing, China.

[4] Department of Interdisciplinary Life Sciences, Research Institute of Wildlife Ecology, Vetmeduni Vienna, Vienna, Austria.

[5] Department of Pharmacology and Therapeutics, College of Medicine & Health Sciences, Khalifa University, Abu Dhabi, United Arab Emirates.

[6] Department of Zoology, King Saud University, Riyadh, Kingdom of Saudi Arabia.

[*] *These authors contributed equally*

[†] These authors jointly supervised this work

Corresponding authors: David Murphy (d.murphy@bristol.ac.uk), Abdu Adem (abdu.adem@ku.ac.ae) and Fernando Alvira-Iraizoz (f.alvirairaizoz@gmail.com)

Supplementary Data

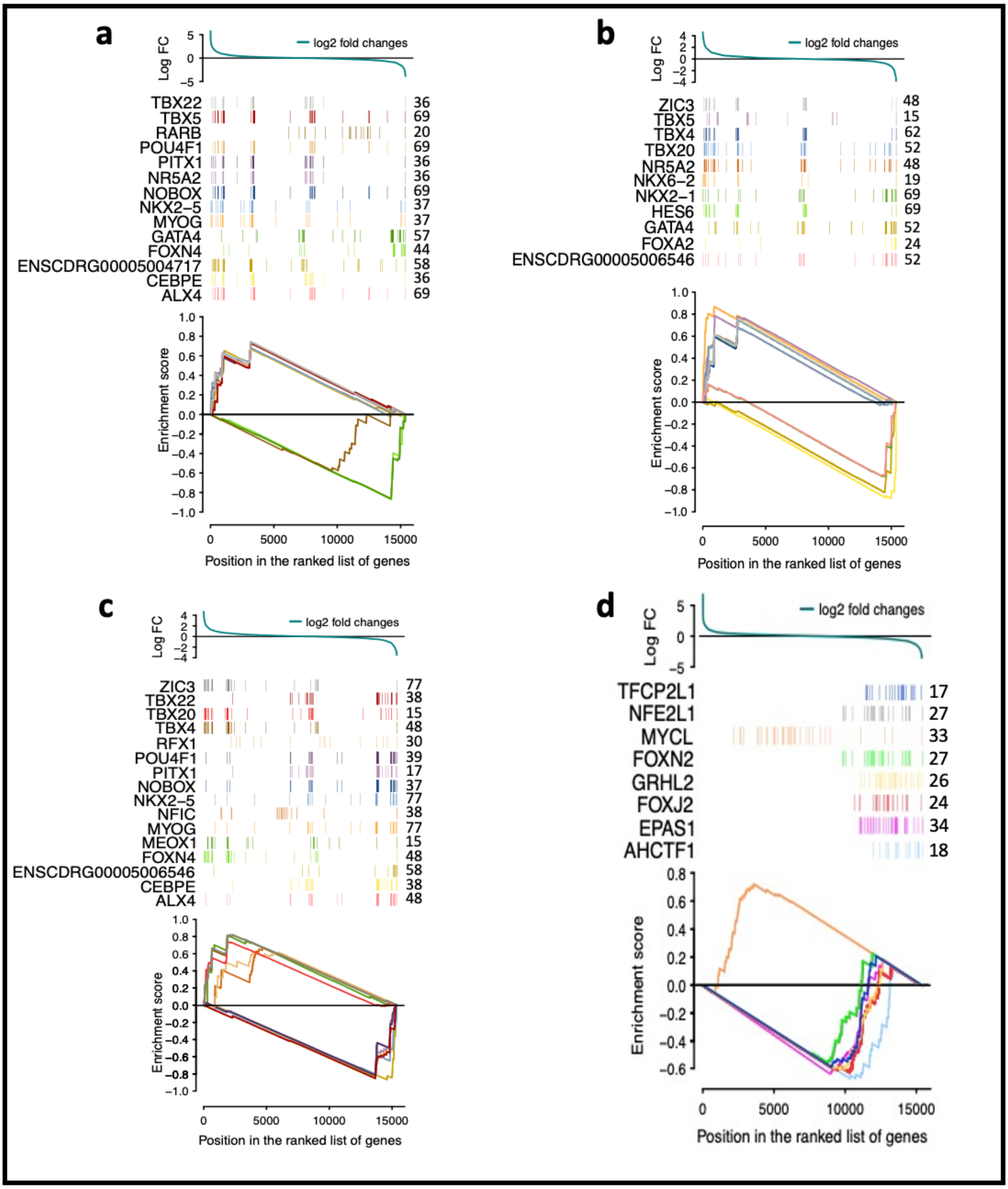

Supplementary Figure SF1. Arabian camel significantly enriched regulons. All significantly enriched transcriptional regulatory networks identified by GSEA2 analysis in camels during dehydration compared to controls**.** The numbers beside the plots note the

number of genes that belong to that specific regulatory network (e.g. TBX22 regulatory network was significantly enriched in dehydrated camels compared to controls and 36 genes of that network are found in this study).**a)** Cortex, dehydration vs. controls. **b)** Cortex, rehydration vs. controls. **c)** Cortex, rehydration vs. dehydration. **d)** Medulla, dehydration vs. controls.

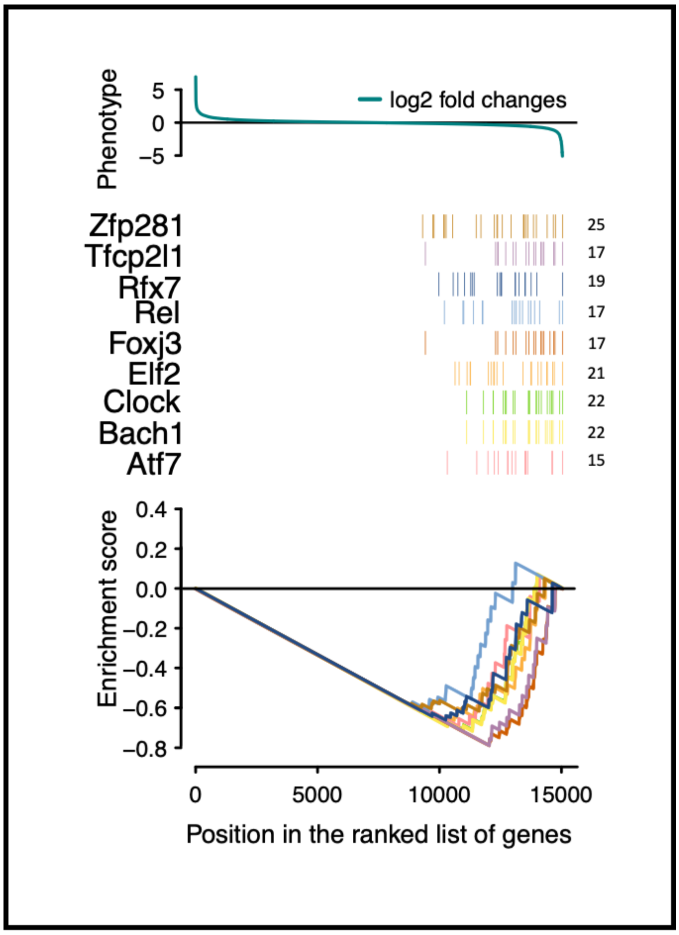

Supplementary Figure SF2. Jerboa significantly enriched regulons. All significantly enriched transcriptional regulatory networks identified by GSEA2 analysis in jerboa during dehydration compared to controls**.** The numbers beside the plots note the number of genes that belong to that specific regulatory network (e.g. Zfp281 regulatory network was significantly enriched in dehydrated jerboas compared to controls and 25 genes of that network are found in this study).

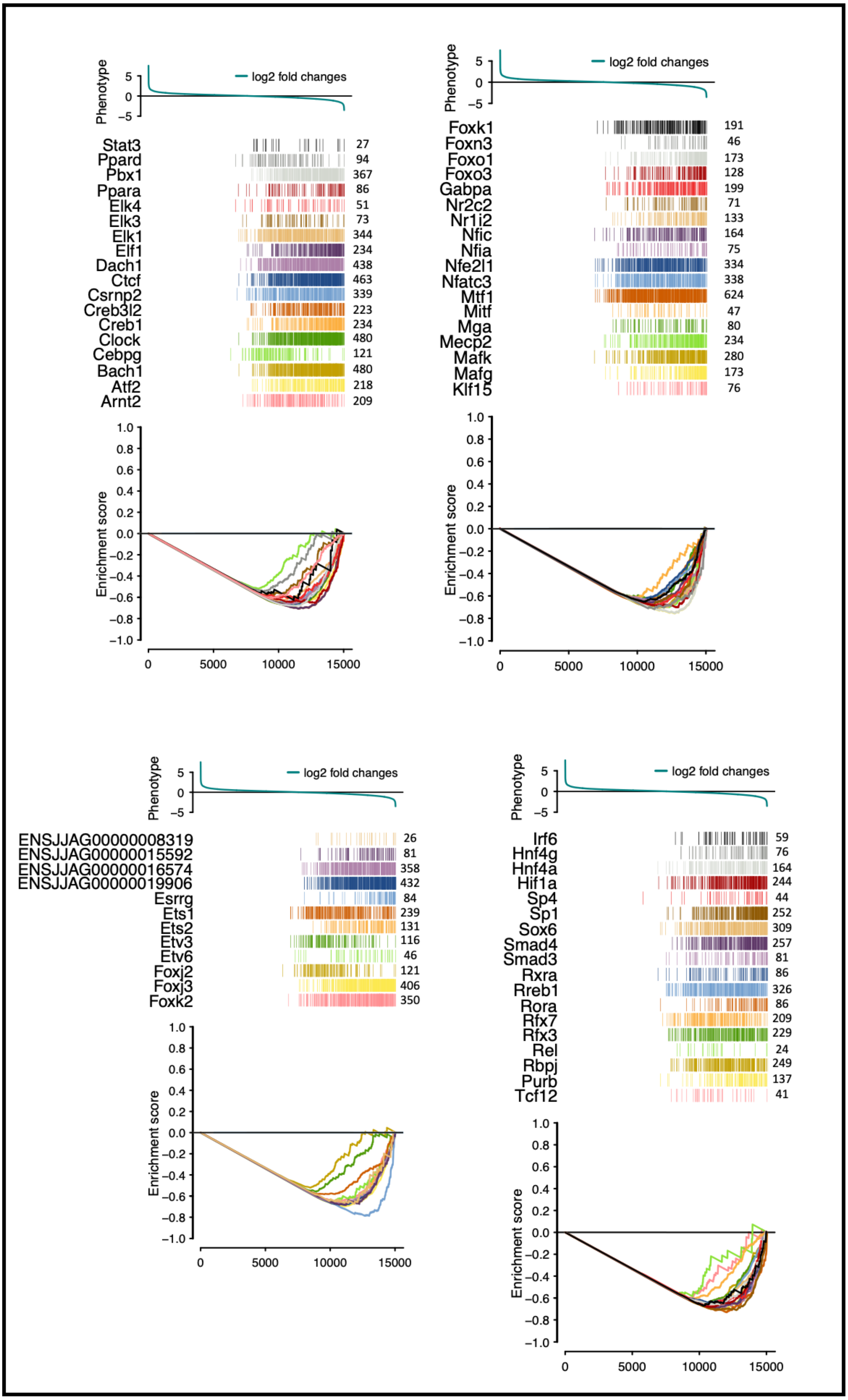
Phenotype
5
0
−5
log2 fold changes
Stat3 27
Ppard 94
Pbx1 367
Ppara 86
Elk4 51
Elk3 73
Elk1 344
Elf1 234
Dach1 438
Ctcf 463
Csrnp2 339
Creb3l2 223
Creb1 234
Clock 480
Cebpg 121
Bach1 480
Atf2 218
Arnt2 209
Enrichment score
1.0
0.8
0.6
0.4
0.2
0.0
−0.2
−0.4
−0.6
−0.8
−1.0
0
5000
10000
15000
Phenotype
log2 fold changes
Foxk1 191
Foxn3 46
Foxo1 173
Foxo3 128
Gabpa 199
Nr2c2 71
Nr1i2 133
Nfic 164
Nfia 75
Nfe2l1 334
Nfatc3 338
Mtf1 624
Mitf 47
Mga 80
Mecp2 234
Mafk 280
Mafg 173
Klf15 76
Enrichment score
Phenotype
log2 fold changes
ENSJJAG00000008319 26
ENSJJAG00000015592 81
ENSJJAG00000016574 358
ENSJJAG00000019906 432
Esrrg 84
Ets1 239
Ets2 131
Etv3 116
Etv6 46
Foxj2 121
Foxj3 406
Foxk2 350
Enrichment score
Phenotype
log2 fold changes
Irf6 59
Hnf4g 76
Hnf4a 164
Hif1a 244
Sp4 44
Sp1 252
Sox6 309
Smad4 257
Smad3 81
Rxra 86
Rreb1 326
Rora 86
Rfx7 209
Rfx3 229
Rel 24
Rbpj 249
Purb 137
Tcf12 41
Enrichment score

Supplementary Figure SF3. Jerboa significantly enriched regulons. All significantly enriched transcriptional regulatory networks identified by GSEA2 analysis in jerboa during rehydration compared to controls**.** The numbers beside the plots note the number of genes that belong to that specific regulatory network (e.g. Stat3 regulatory network was significantly enriched in rehydrated jerboas compared to controls and 27 genes of that network are found in this study).

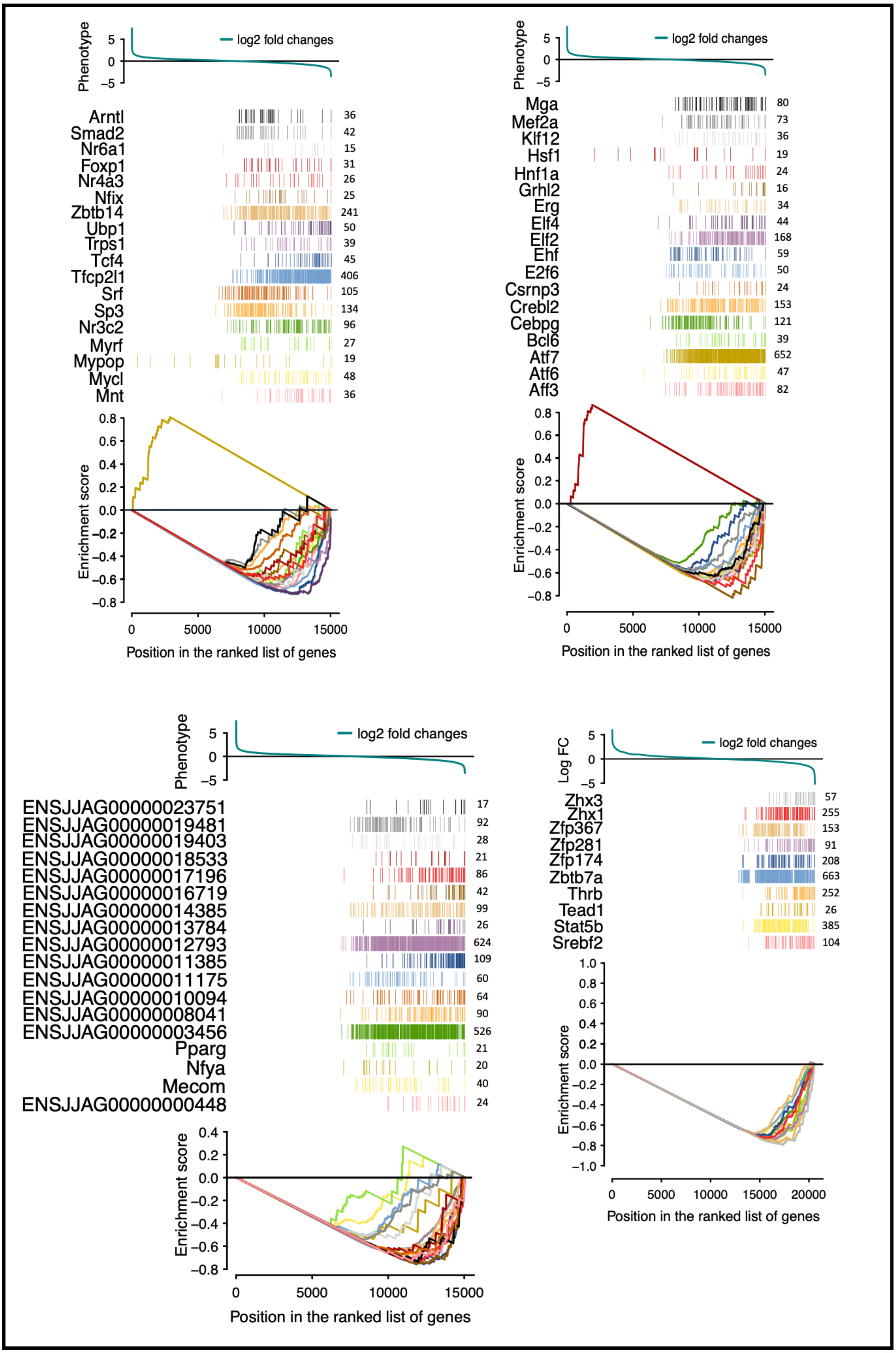

Phenotype
log2 fold changes
Arntl 36
Smad2 42
Nr6a1 15
Foxp1 31
Nr4a3 26
Nfix 25
Zbtb14 241
Ubp1 50
Trps1 39
Tcf4 45
Tfcp2l1 406
Srf 105
Sp3 134
Nr3c2 96
Myrf 27
Mypop 19
Mycl 48
Mnt 36
Enrichment score
Position in the ranked list of genes
Phenotype
log2 fold changes
Mga 80
Mef2a 73
Klf12 36
Hsf1 19
Hnf1a 24
Grhl2 16
Erg 34
Elf4 44
Elf2 168
Ehf 59
E2f6 50
Csrnp3 24
Crebl2 153
Cebpg 121
Bcl6 39
Atf7 652
Atf6 47
Aff3 82
Enrichment score
Position in the ranked list of genes
Phenotype
log2 fold changes
ENSJJAG00000023751 17
ENSJJAG00000019481 92
ENSJJAG00000019403 28
ENSJJAG00000018533 21
ENSJJAG00000017196 86
ENSJJAG00000016719 42
ENSJJAG00000014385 99
ENSJJAG00000013784 26
ENSJJAG00000012793 624
ENSJJAG00000011385 109
ENSJJAG00000011175 60
ENSJJAG00000010094 64
ENSJJAG00000008041 90
ENSJJAG00000003456 526
Pparg 21
Nfya 20
Mecom 40
ENSJJAG00000000448 24
Enrichment score
Position in the ranked list of genes
Log FC
log2 fold changes
Zhx3 57
Zhx1 255
Zfp367 153
Zfp281 91
Zfp174 208
Zbtb7a 663
Thrb 252
Tead1 26
Stat5b 385
Srebf2 104
Enrichment score
Position in the ranked list of genes

Supplementary Figure SF3 (cont.). Jerboa significantly enriched regulons.

Rehydration compared to controls**.**

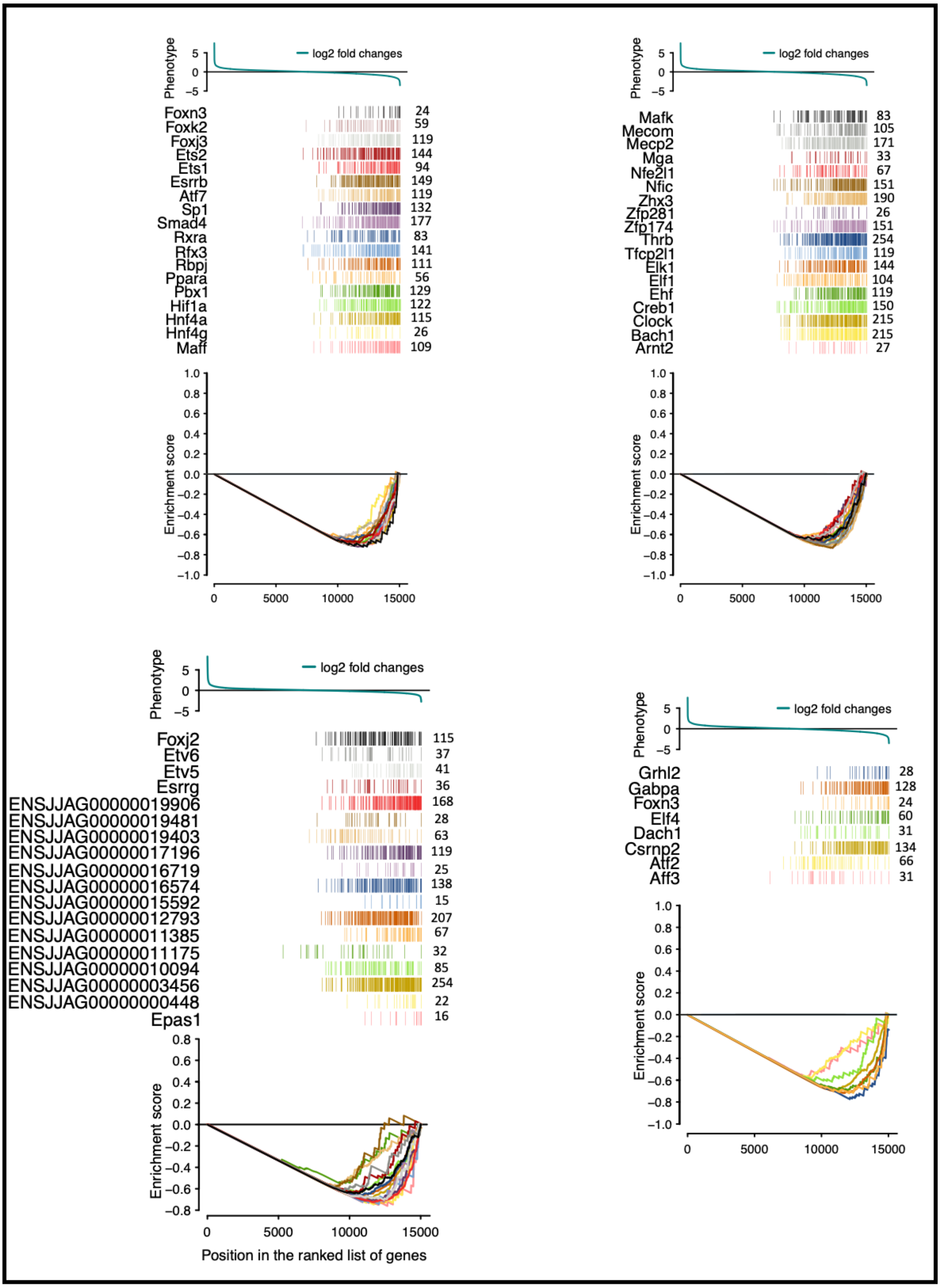

Supplementary Figure SF4. All significantly enriched transcriptional regulatory networks identified by GSEA2 analysis in jerboa during rehydration compared to

dehydration. The numbers beside the plots note the number of genes that belong to that specific regulatory network (e.g. Stat3 regulatory network was significantly enriched in rehydrated jerboas compared to controls and 27 genes of that network are found in this study).

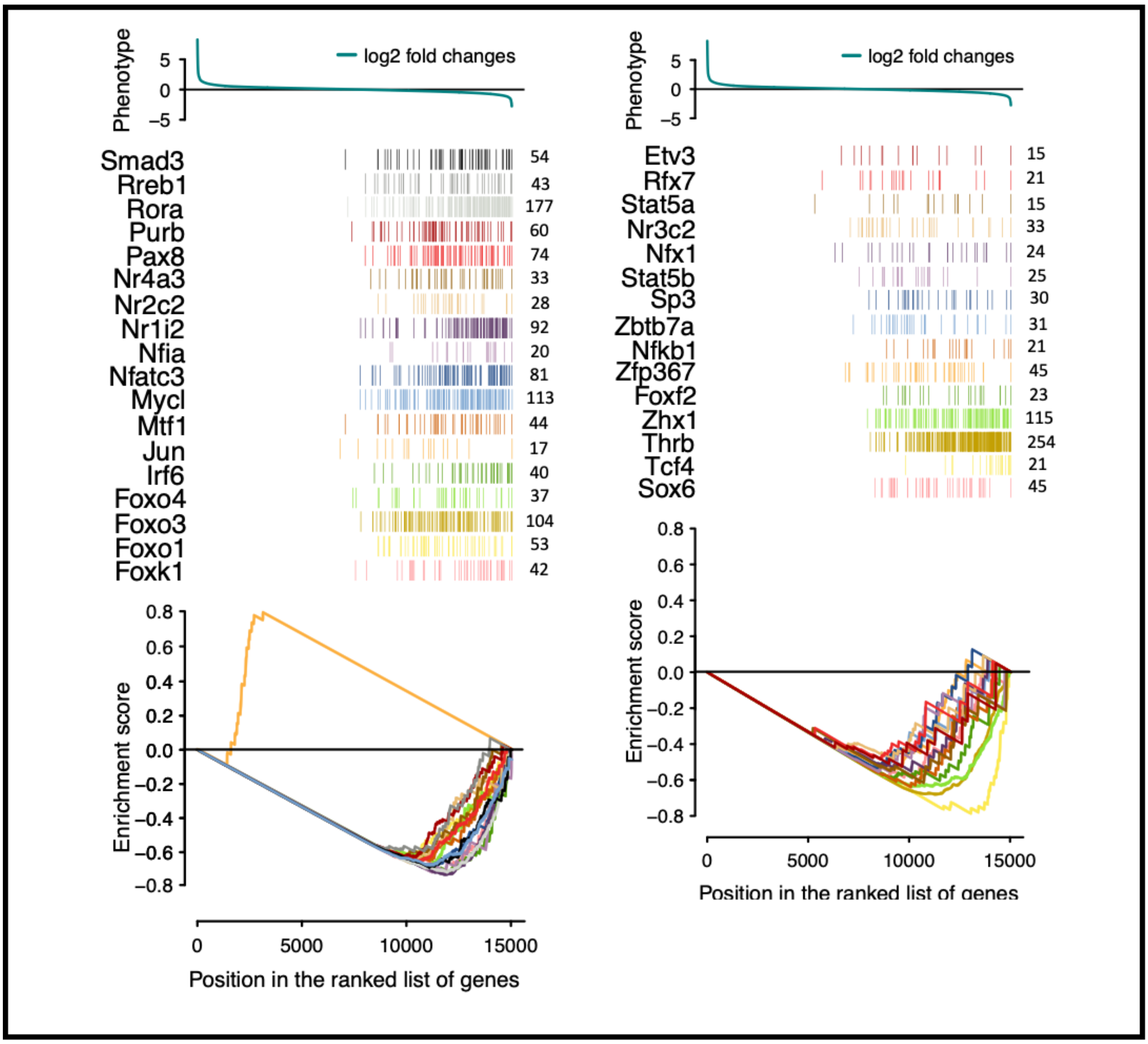

Supplementary Figure SF4 (cont.). Rehydration compared to dehydration.

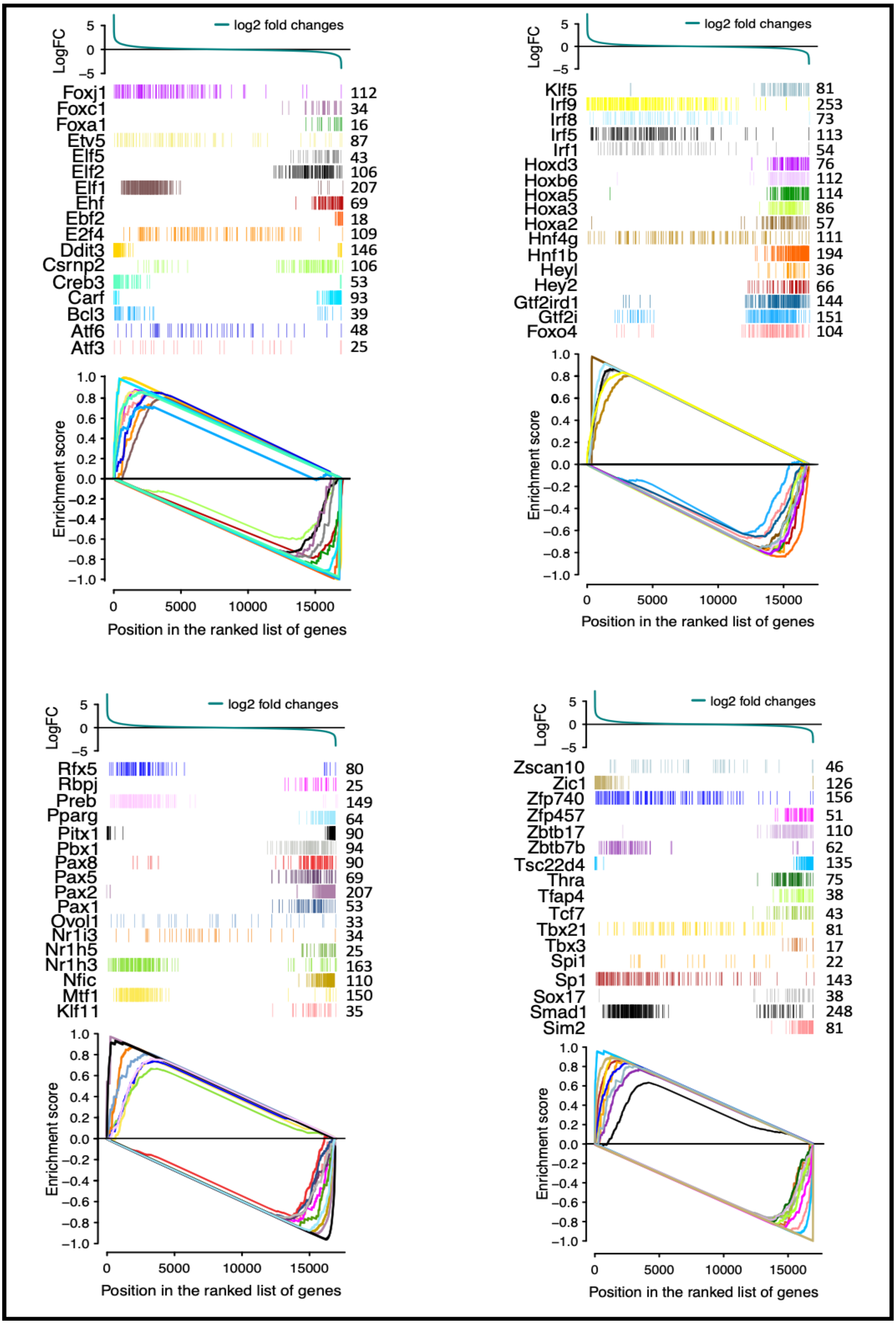
log2 fold changes
LogFC
Foxj1 112
Foxc1 34
Foxa1 16
Etv5 87
Elf5 43
Elf2 106
Elf1 207
Ehf 69
Ebf2 18
E2f4 109
Ddit3 146
Csrnp2 106
Creb3 53
Carf 93
Bcl3 39
Atf6 48
Atf3 25
Enrichment score
Position in the ranked list of genes
Klf5 81
Irf9 253
Irf8 73
Irf5 113
Irf1 54
Hoxd3 76
Hoxb6 112
Hoxa5 114
Hoxa3 86
Hoxa2 57
Hnf4g 111
Hnf1b 194
Heyl 36
Hey2 66
Gtf2ird1 144
Gtf2i 151
Foxo4 104
Rfx5 80
Rbpj 25
Preb 149
Pparg 64
Pitx1 90
Pbx1 94
Pax8 90
Pax5 69
Pax2 207
Pax1 53
Ovol1 33
Nr1i3 34
Nr1h5 25
Nr1h3 163
Nfic 110
Mtf1 150
Klf11 35
Zscan10 46
Zic1 126
Zfp740 156
Zfp457 51
Zbtb17 110
Zbtb7b 62
Tsc22d4 135
Thra 75
Tfap4 38
Tcf7 43
Tbx21 81
Tbx3 17
Spi1 22
Sp1 143
Sox17 38
Smad1 248
Sim2 81

Supplementary Figure SF5. All significantly enriched transcriptional regulatory networks identified by GSEA2 analysis in olive mice during dehydration compared to controls. The numbers beside the plots note the number of genes that belong to that specific regulatory network (e.g. Foxj1 regulatory network was significantly enriched in rehydrated jerboas compared to controls and 112 genes of that network are found in this study).

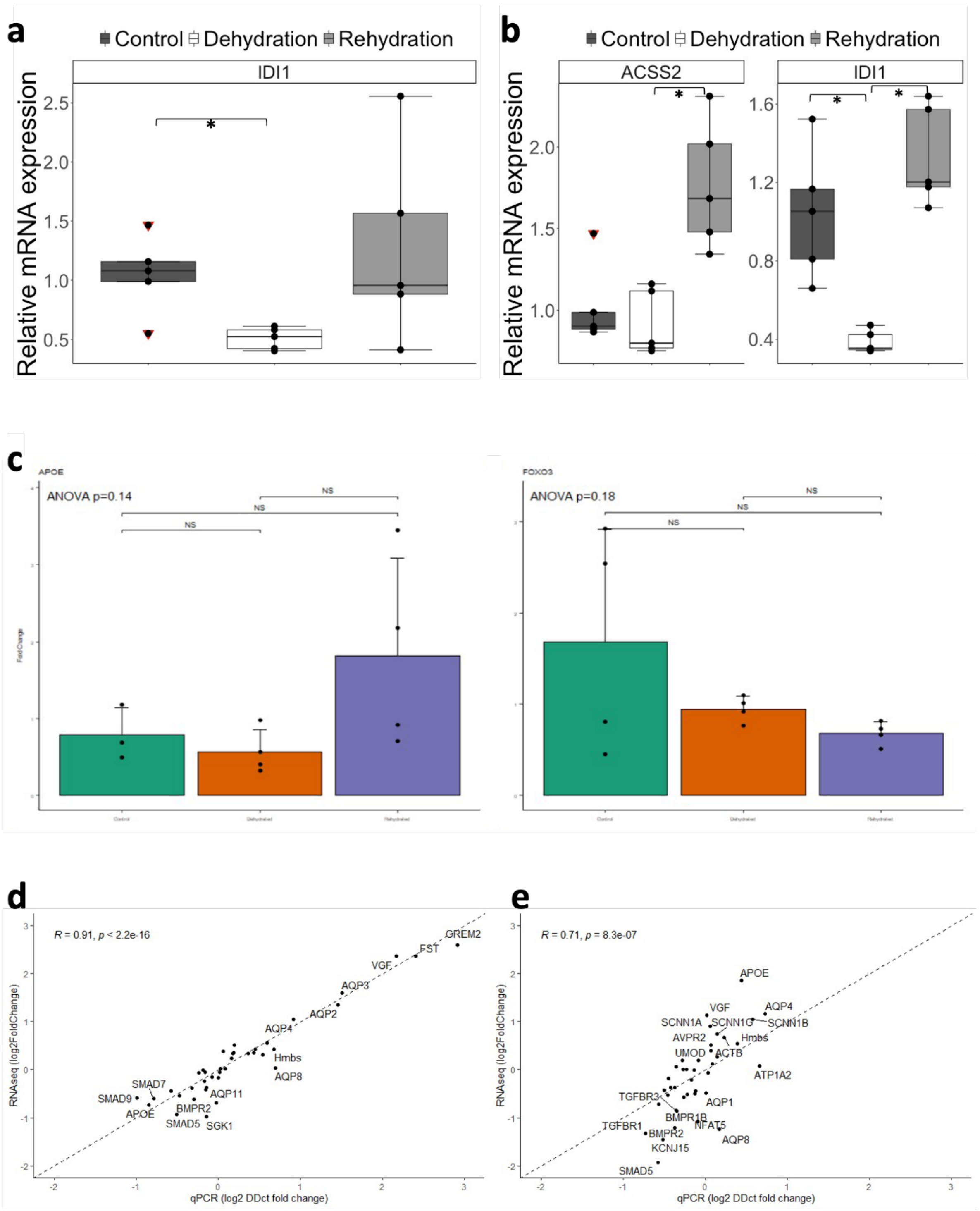

Supplementary Figure SF6. Validation of SREBP1 and SREBP2 target genes. a) Relative changes in gene expression of IDI1 in the Arabian camel kidney cortex after chronic dehydration and acute rehydration compared to controls. b) Relative changes in

gene expression of IDI1 and ACSS2 in the Arabian camel kidney medulla after chronic dehydration and acute rehydration compared to controls. c) Relative changes in gene expression of APOE and FOXO3 in the jerboa kidney after chronic dehydration and acute rehydration compared to controls. Comparison of the means by one-way ANOVA (Tukey’s post hoc correction). The boxplots are presented with the S.E.M (n = 5), centre lines show median, box edges delineate 25th and 75th percentiles and bars extend to minimum and maximum values. Individual data points represent biologically independent samples and data points within red triangles denote outliers, all the outliers highlighted were included for the statistical analyses. d) and e) show the comparison of the fold change after dehydration (d) and rehydration (e) between qPCR and RNAseq for all genes measured that shows good correlation between the two techniques.

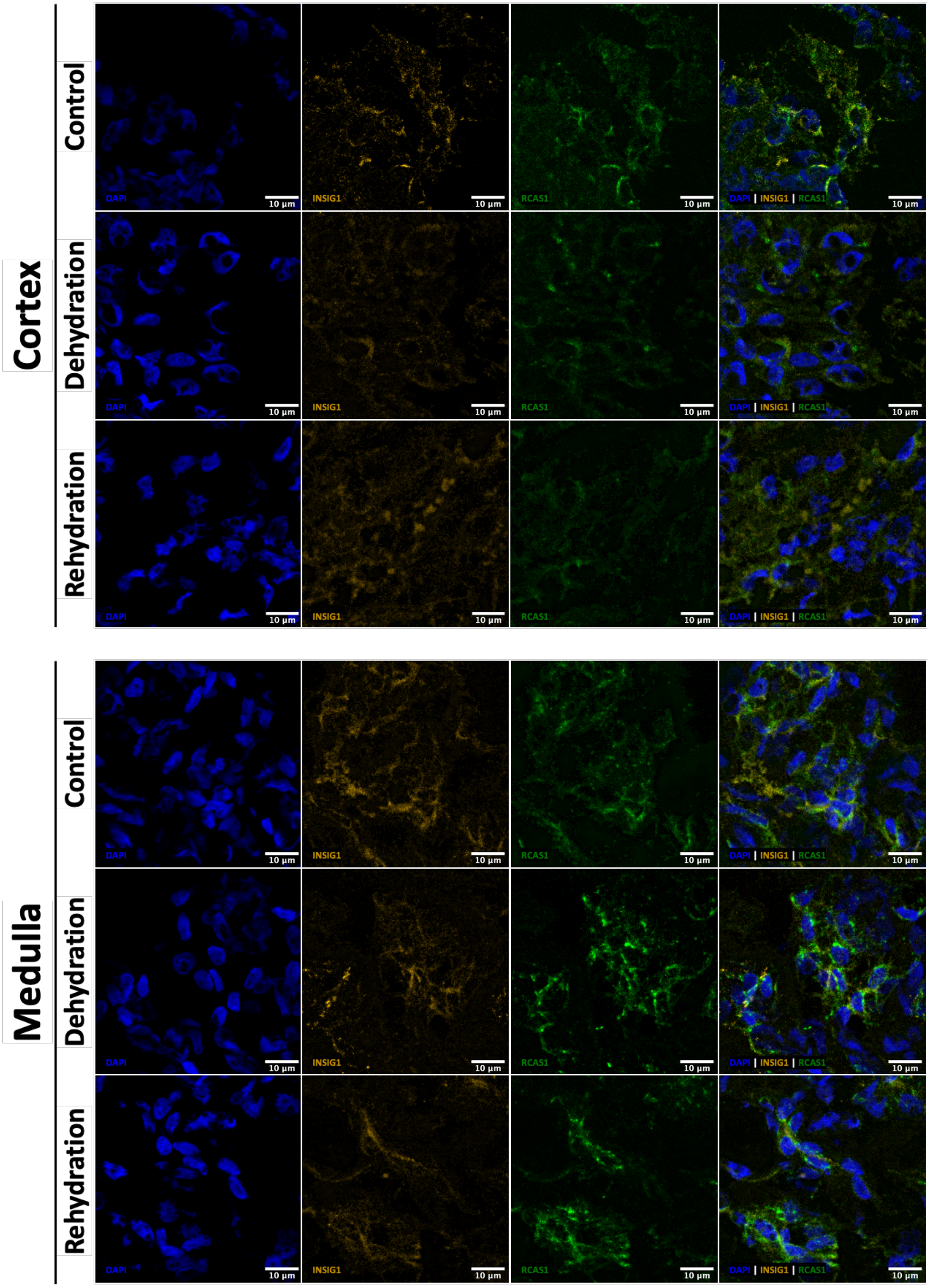

Supplementary Figure SF7. Cellular localization of INSIG1 in the kidney of the one-humped Arabian camel during chronic dehydration and acute rehydration.

Immunofluorescence staining of Insulin Induced Gene 1 (INSIG1) in camel kidney cortex and medulla sections from controls, dehydrated and rehydrated animals. Images are representative of several cross sections of each kidney compartment. Localization of INSIG1 (yellow) is shown relative to the nuclei (DAPI, blue, 1st column) and the Golgi apparatus (RCAS1, green, 3rd column) in the overlap images (4th column). Scale bars, 10 μm.

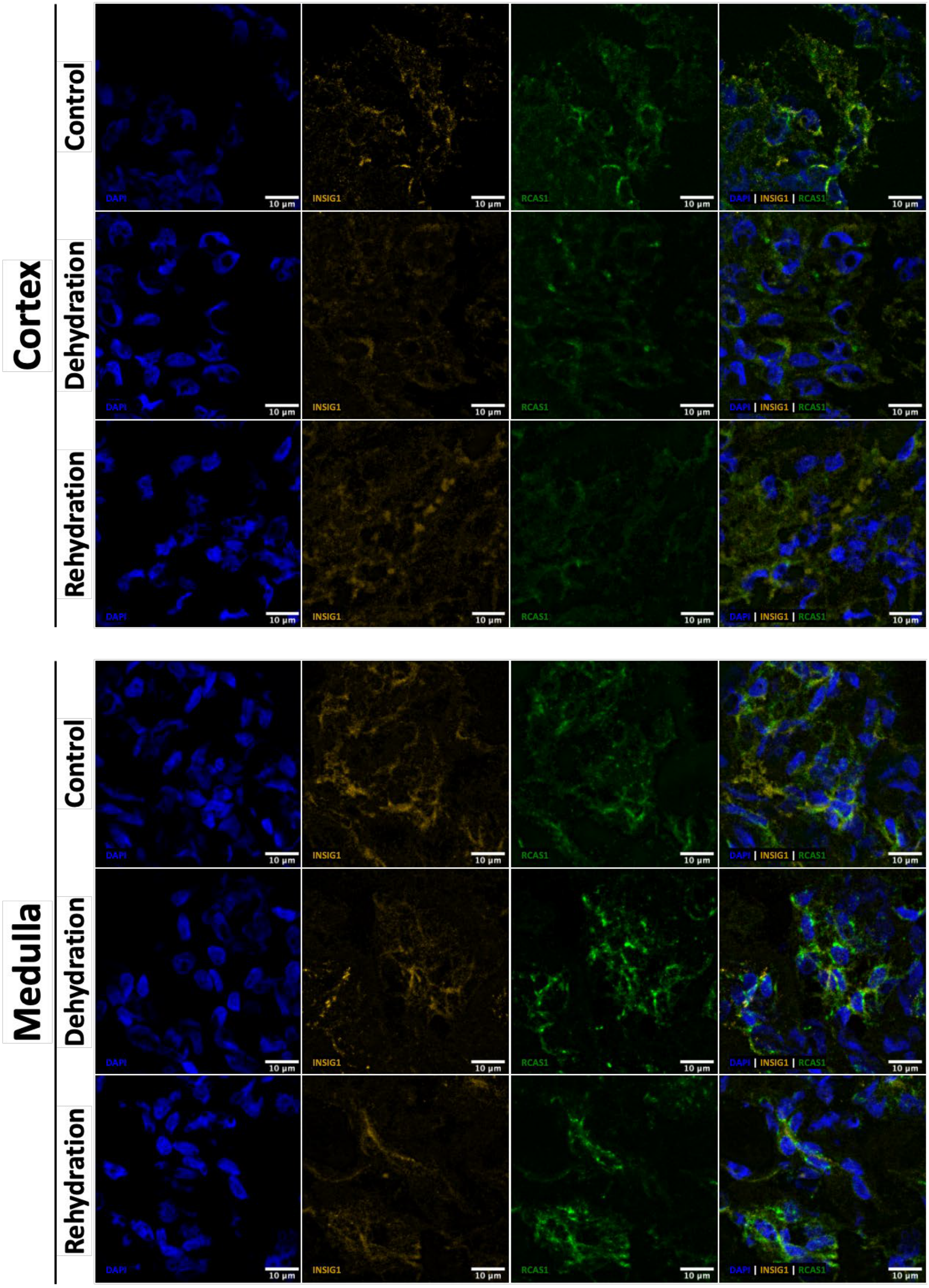

Supplementary Figure SF8. Cellular localization of INSIG1 in the kidney of the jerboas during chronic dehydration and acute rehydration. Immunofluorescence

staining of Insulin Induced Gene 1 (INSIG1) in jerboa whole kidney sections from controls, dehydrated and rehydrated animals. Images are representative of several cross sections of the kidney. Localization of INSIG1 (yellow) is shown relative to the nuclei (DAPI, blue, 1st column) and the Golgi apparatus (RCAS1, green, 3rd column) in the overlap images (4th column). Scale bars, 10 µm.

Primer sequences for RT-qPCR validation					
Camel			Jerboa		
INSIG1	Fwd CTTGGGATCACCATCGCTTT	Rev CAGTCCACATACATCACATGGC	*INSIG1*	Fwd CTCTTTCCCGACGAGGTGAT	Rev GGCCCACTCTCTCTTGAACT
SREBF1	Fwd GGCTCAGGTGACTCAGCTAT	Rev GAGAACTCCCTGTCCCCATC	*SREBF1*	Fwd GACCGACATCGAAGACATGC	Rev GCATAGGGGGCATCAAACAG
SREBF2	Fwd TACCGCAAGGCTACTTGTCT	Rev TAAGGGCTGCTTCCTCTACG	*SREBF2*	Fwd ACCGAGCACACTGATTGAGA	Rev AGGGACAGTGCTATGCTCAG
IDI1	Fwd ACGTGTCCTGATTTCCGTCA	Rev ACCCAGCCAATGTCTAACCA	*APOE*	Fwd TGGGCCGTTTCTGGGATTAC	Rev ATCAGCACCGTCAGTTCCTG
SQLE	Fwd AACGCCATTGGAGGCAAGTA	Rev CCCCATCCTCTCACCAGTTC	*FOXO3*	Fwd TTCATCTCAGAGCTGGGTGC	Rev GTGTCAGTTTGAGGGTCTGCT
PPIA	Fwd ACCACCAGACCATTCCTTCT	Rev TATGGAACCCCGAAAACTGC	*PPIA*	Fwd CATCTGCACTGCCAAGACTG	Rev TTCCTAGACCCAAGGCGTTC

Supplementary Table ST1. Primer sets used for RT-qPCR validation
