# Supplementary material for: Reconstruction of the transcriptional regulatory networks in the kidney of desert-adapted species
Source: Commun Biol. 2025 Nov 28;8:1719. doi: 10.1038/s42003-025-09124-2 (PMC12663168; doi:10.1038/s42003-025-09124-2)
Supplement: Supplementary file 5 — Reporting Summary [file 42003_2025_9124_MOESM5_ESM.pdf]

nature portfolio

Corresponding author(s): David Murphy, Abdu Adem, Fernando Alvira-Iraizoz

Last updated by author(s): Sep 17, 2025

Reporting Summary

Nature Portfolio wishes to improve the reproducibility of the work that we publish. This form provides structure for consistency and transparency in reporting. For further information on Nature Portfolio policies, see our Editorial Policies and the Editorial Policy Checklist.

Statistics

For all statistical analyses, confirm that the following items are present in the figure legend, table legend, main text, or Methods section.

n/a	Confirmed	
☐	☒	The exact sample size (*n*) for each experimental group/condition, given as a discrete number and unit of measurement
☐	☒	A statement on whether measurements were taken from distinct samples or whether the same sample was measured repeatedly
☐	☒	The statistical test(s) used AND whether they are one- or two-sided<br>*Only common tests should be described solely by name; describe more complex techniques in the Methods section.*
☒	☐	A description of all covariates tested
☐	☒	A description of any assumptions or corrections, such as tests of normality and adjustment for multiple comparisons
☐	☒	A full description of the statistical parameters including central tendency (e.g. means) or other basic estimates (e.g. regression coefficient) AND variation (e.g. standard deviation) or associated estimates of uncertainty (e.g. confidence intervals)
☐	☒	For null hypothesis testing, the test statistic (e.g. *F*, *t*, *r*) with confidence intervals, effect sizes, degrees of freedom and *P* value noted<br>*Give P values as exact values whenever suitable.*
☒	☐	For Bayesian analysis, information on the choice of priors and Markov chain Monte Carlo settings
☒	☐	For hierarchical and complex designs, identification of the appropriate level for tests and full reporting of outcomes
☒	☐	Estimates of effect sizes (e.g. Cohen's *d*, Pearson's *r*), indicating how they were calculated

Our web collection on statistics for biologists contains articles on many of the points above.

Software and code

Policy information about availability of computer code

Data collection	We did not use commercial, open source or custom code to collect data in this study
Data analysis	All software and code used to analyze the RNAseq data has been previously described in the literature and are common, well-established tools used in omics studies. These can be found in Giorello et al 2014 and 2018, Alvira-Iraizoz et al 2019 and Gillard et al 2023; these 3 papers are referenced in the manuscript, and a brief description can be found in the methods section of this manuscript. The scripts used to run the RTN analyses are described in Fletcher et al and Castro et al. Also described in the Methods Section.

For manuscripts utilizing custom algorithms or software that are central to the research but not yet described in published literature, software must be made available to editors and reviewers. We strongly encourage code deposition in a community repository (e.g. GitHub). See the Nature Portfolio guidelines for submitting code & software for further information.

Data

Policy information about availability of data

All manuscripts must include a data availability statement. This statement should provide the following information, where applicable:

- Accession codes, unique identifiers, or web links for publicly available datasets
- A description of any restrictions on data availability
- For clinical datasets or third party data, please ensure that the statement adheres to our policy

The transcriptomic data underlying these analyses, including raw FASTQ files, bulk RNAseq counts, DESeq2 data and project metadata, were deposited in NCBI's

Gene Expression Omnibus (GEO) or NCBI Sequence Read Archive (SRA) as part of a previous publications. One-humped Arabian camel data is accessible through GEO Series accession number GSE173683 at https://www.ncbi.nlm.nih.gov/geo/query/acc.cgi?acc=GSE173683. Jerboa data is accessible through GEO Series accession number GSE225470 at https://pmc.ncbi.nlm.nih.gov/articles/PMC10470305/. Olive mouse data was deposited in NCBI SRA, and reads are available under BioProject accession number PRJNA471316. Reference transcriptome assembly is available through Dryad at https://doi.org/10.5061/dryad.7nh50k7. SREBP1 and SREBP2 target genes lists were downloaded from ChIP-X database which is freely available at http://amp.pharm.mssm.edu/lib/chea.jsp.

Research involving human participants, their data, or biological material

Policy information about studies with human participants or human data. See also policy information about sex, gender (identity/presentation), and sexual orientation and race, ethnicity and racism.

Reporting on sex and gender	N/A
Reporting on race, ethnicity, or other socially relevant groupings	N/A
Population characteristics	N/A
Recruitment	N/A
Ethics oversight	N/A

Note that full information on the approval of the study protocol must also be provided in the manuscript.

Field-specific reporting

Please select the one below that is the best fit for your research. If you are not sure, read the appropriate sections before making your selection.

☒ Life sciences ☐ Behavioural & social sciences ☐ Ecological, evolutionary & environmental sciences

For a reference copy of the document with all sections, see nature.com/documents/nr-reporting-summary-flat.pdf

Life sciences study design

All studies must disclose on these points even when the disclosure is negative.

Sample size	We used data previously published to run our analyses. All information regarding samples size can be found in Giorello et al 2014 and 2018, Alvira-Iraizoz et al 2019 and Gillard et al 2023. These paper are freely available online and a full reference can be found in the manuscript.
Data exclusions	Similarly to the comment above, we used data previously published to run our analyses. All information regarding samples size can be found in Giorello et al 2014 and 2018, Alvira-Iraizoz et al 2019 and Gillard et al 2023. These paper are freely available online and a full reference can be found in the manuscript.
Replication	This work was performed using samples from a non-model organisms (camels, jerboas and Olive mice) so replication of the studies was not possible due to the impossibility to perform additional field work.
Randomization	The animals used in these experiments were randomly divided into groups or randomly capture in their environment. Detailed information can be found in Giorello et al 2014 and 2018, Alvira-Iraizoz et al 2019 and Gillard et al 2023. These paper are freely available online and a full reference can be found in the manuscript.
Blinding	The investigators were not blinded during sample collection. An alphanumeric coding system was used post extraction, so investigators performing and analysing RNA sequencing were blinded.

Reporting for specific materials, systems and methods

We require information from authors about some types of materials, experimental systems and methods used in many studies. Here, indicate whether each material, system or method listed is relevant to your study. If you are not sure if a list item applies to your research, read the appropriate section before selecting a response.

Materials & experimental systems

n/a	Involved in the study	
☐	☒	Antibodies
☒	☐	Eukaryotic cell lines
☒	☐	Palaeontology and archaeology
☐	☒	Animals and other organisms
☒	☐	Clinical data
☒	☐	Dual use research of concern
☒	☐	Plants

Methods

n/a	Involved in the study	
☒	☐	ChIP-seq
☒	☐	Flow cytometry
☒	☐	MRI-based neuroimaging

Antibodies

Antibodies used	Primary antibodies anti-mouse SREBP-1 (diluted 1:50 in 1% BSA PBS-t; Santa Cruz Biotechnology, Inc., sc-365513, Lot L0420), anti-mouse SREBP-2 (diluted 1:50 in 1% BSA PBS-t; Santa Cruz Biotechnology, Inc., sc-13552, Lot J0820) and anti-mouse INSIG-1 (diluted 1:50 in 1% BSA PBS-t; Santa Cruz Biotechnology, Inc., sc-390504, Lot L2116), anti-rabbit RCAS1 as Golgi marker (diluted 1:150 in 1% BSA PBS-t; Cell Signalling Technology, Inc., D6P5J, Lot:1)
Validation	Unfortunately, antibodies were primarily design for use in human samples. We did run internal validation by series of optimization.

Animals and other research organisms

Policy information about studies involving animals; ARRIVE guidelines recommended for reporting animal research, and Sex and Gender in Research

Laboratory animals	This study did not involved laboratory animals.
Wild animals	Only Olive mice were wild, details of the procedures regarding wild animals can be found in Giorello et al 2014 and 2018. These paper are freely available online and a full reference can be found in the manuscript.
Reporting on sex	All details can be found in Giorello et al 2014 and 2018, Alvira-Iraizoz et al 2019 and Gillard et al 2023. These paper are freely available online and a full reference can be found in the manuscript.
Field-collected samples	All details can be found in Giorello et al 2014 and 2018, Alvira-Iraizoz et al 2019 and Gillard et al 2023. These paper are freely available online and a full reference can be found in the manuscript.
Ethics oversight	The camel project was approved by the Animal Ethics Committee of the United Arab Emirates University and the University of Bristol Animal Welfare and Ethical Review Board.<br><br>The jerboa project was approved by the Animal Ethics Committee of the United Arab Emirates University and the University of Bristol Animal Welfare and Ethical Review Board.<br><br>The Olive mice project was approved by the Ethics Committee of the Fondo Nacional de Ciencia y Tecnología (FONDECYT, Chile) and the Ethics Committee of the Universidad Austral de Chile (UACh, Chile), as part of the review process for the Fondecyt Research Grant 1110737

Note that full information on the approval of the study protocol must also be provided in the manuscript.

Plants

Seed stocks	N/A
Novel plant genotypes	N/A
Authentication	N/A
